# Particle Image Velocimetry of 3D-Printed Anatomical Blood Vascular Models Affected by Atherosclerosis

**DOI:** 10.3390/ma16031055

**Published:** 2023-01-25

**Authors:** Arkadiusz Antonowicz, Krzysztof Wojtas, Łukasz Makowski, Wojciech Orciuch, Michał Kozłowski

**Affiliations:** 1 Eurotek International Ltd., Skrzetuskiego 6, 02-726 Warsaw, Poland; 2 Faculty of Chemical and Process Engineering, Warsaw University of Technology, Waryńskiego 1, 00-645 Warsaw, Poland; 3 Department of Cardiology and Structural Heart Diseases, Medical University of Silesia, Ziołowa 47, 40-635 Katowice, Poland

**Keywords:** particle image velocimetry, micro-PIV, 3D printing, anatomical vascular models, atherosclerosis

## Abstract

Improvements in the diagnosis and treatment of cardiovascular diseases facilitate a better understanding of the ongoing process. The study of biomedical fluid dynamics using non-intrusive visualizing methods on a micro-scale has become possible using a proper 3D printing process. The computed tomography scan of a patient with atherosclerosis was processed, and a 3D-printed artery with an inlet diameter of 4.2 mm was developed and measured using three different constant flow rates. To mimic blood, a solution of glycerin and water was used. The procedure to obtain a proper 3D-printed model using low-force stereolithography technology with high-quality optical access usable for PIV was described and discussed. The paper presents the results of PIV as multi-stitched, color-coded vector maps from the axis cross section along the whole 3D-printed model. The obtained data allowed a resolution of 100 × 100 µm per single vector to be achieved. Furthermore, the results of the stitched 16 base images of the artery and the 3D-printed model prepared were included. The results of this study show that 3D prints allow for the creation of the desired geometry and can be used to investigate severe pathologies of the human circulatory system. The strengths and weaknesses of this methodology were discussed and compared to other techniques used to obtain transparent objects.

## 1. Introduction

Studies of flows within the human body, especially those within the circulatory system, have attracted significant interest in recent years. Computational fluid dynamics plays an important role in the modeling and simulation for such research. New knowledge obtained from this research field may improve the prevention, detection, or even treatment of dangerous pathologies occurring in the human body. However, even the best simulations require accurate validation, driven largely by the development of advanced measurement techniques, combined with the constant endeavor to better understand the complex processes occurring within the human body.

Many cardiovascular diseases are already of wide interest for in vitro studies. These include stenosis [1], mitral regurgitation [2], hemolysis [3], and paravalvular leaks [4,5] (which all may occur following heart valve replacement surgery), as well as pathologies affecting venous valves [6] or stents [7].

One of the most useful imaging measurement techniques used to validate in silico findings is particle image velocimetry (PIV). This experimental fluid dynamics tool has some limitations and demands that require well-prepared measurements in order to obtain valuable results, especially in micro-scale structures [8]. In micro-PIV, the depth of correlation is another important parameter. Increasing the depth of focus leads to increasing the importance of out-of-focus particles in determining the value of the velocity, while maintaining the thinnest-possible measuring volume [9]. To meet the requirements of the PIV technique, a first choice would be a silicone model [10], though over time, 3D printing techniques have been used to improve the interior of the cast models [11]. Such models provide good results, but their preparation is time-consuming, and they are relatively expensive to produce. Hydrogel phantoms possess transparency and parameters that are required to work with imaging-based measurement systems [12]. A review of different kinds of hydrogel groups was completed by [13], outlining different advantages and disadvantages. Another obvious choice is 3D printing, especially considering the rapid development of this technology and used materials, which can allow reliable and inexpensive models to be generated. One example of this is Clear Resin from Formlabs, whose products can be directly used for PIV measurements without the requirement of other materials or supports. The guidelines developed in our study make it possible to make a good-quality 3D-printed object without using an external company [14].

The physical properties of the material itself, other than transparency, are also important. Since the PIV system includes a laser, the material must be insensitive to the 532 nm high-pulse energy laser beam focused into a thin, short line. Additionally, it should offer resistance to some chemical reagents. The problem of the deposition of seed particles on the inner walls of the measured models also cannot be ignored.

Another thing to consider is the rheological behavior of blood. Although it is possible to use blood itself in the measurements [15,16,17], it has limitations and it is even more difficult to use; therefore, to reproduce the viscosity of blood, a glycerin–water solution is usually used. A proper refractive index is also necessary and can be obtained, e.g., by adding sodium iodide salt [18], thereby enabling proper PIV measurements, while considering the actual behavior of blood in the studied system.

This study focuses on experimental work that is used to investigate the possibility of using 3D printing to measure complex geometries with mechanical inaccessibility to long geometry data, especially to investigate the benefits and losses of using the LFS technique. Qualitative work was conducted [19] but with the use of the PolyJet technique. Similar work was conducted by [20], but without PIV results, especially micro-PIV, which has more restrictions. For these reasons, the main purpose behind this study is to develop 3D printing procedures with 2D- and stereo-micro-PIV/PIV that are most useful for various biomedical objects, especially where movable parts are included, e.g., a heart valve, which eliminates the possibility of using non-solid materials.

## 2. Materials and Methods

### 2.1. Printed Model

**Figure 1:**
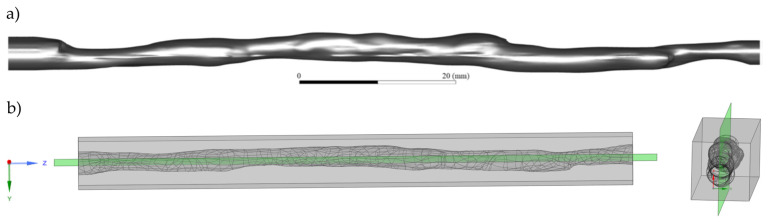
Geometry of the coronary artery: (**a**) side view and (**b**) model prepared for 3D printing with the location of the laser plane relative to the geometry.

As mentioned in the previous section, the material that can be used to print 3D models under the PIV system must meet several requirements. Before proceeding with the selection of the material, the following general assumptions should be made:

- The geometry of the printed model must correspond to the geometry obtained by the computer tomography (CT) scan of the patient with atherosclerosis. The model of the artery was created based on cardiac CT data with contrast. ECG-gated CT was performed on a 256-slice Somatom Siemens CT scanner per the standard acquisition protocol. DICOM images were exported to 3D Slicer software v4.11 (open source). End-diastolic images were selected for further processing. First, the ‘mask volume’ effect was applied to the master volume to exclude extracardiac structures. Second, the ‘local threshold’ effect was used to create two segments: one that contained all contrast-filled cavities (heart chambers and coronary arteries) and another that contained all other tissues. A 3D model of coronary arteries was rendered. Smoothing algorithms were applied. The quality of segmentation was checked. The left anterior descending coronary artery was manually removed from the dataset and exported in .stl format for further processing. The model represents the left anterior descending coronary artery (Figure 1). The model of the artery was created based on anonymized cardiac CT data that were already available in the hospital database. Since no details that could lead to the potential identification of the subject were provided, no informed consent was required. We considered the geometries of arteries with a diameter of about 4 mm and a length of about 100 mm. The same model was also used as input to a computational fluid dynamics program to validate the results.
- Images obtained using the detection part of the PIV system from the inside of the model must be free from distortions that arise on the curves of the walls. There are two scenarios to consider:
  ○ Printed geometries have inner and outer walls with irregular shapes. Thus, we should use a rectangular tank with transparent walls, within which the test object would be placed. The fluid that would be used to measure the flows inside the tested object and the fluid that would be used to fill the space inside the tank and around the test object must match the refractive index of the 3D printing material.
  ○ Printed geometries have inner walls with irregular shapes, but outer walls are flat and have a shape similar to a cuboid. The fluid that would be used to measure the flows inside the test object has to still match the refractive index of the 3D printing material. In addition, the outer walls through which the photos for the PIV analysis would be taken have to be flat and smooth. The dullness of these walls makes measurements practically impossible.
- Transparency, which is of course the overriding parameter. A few years ago, the best transparent 3D prints offered no opportunities for taking optical measurements due to the matte and milky texture of the printout.
- Insensitivity in contact with the tested medium. Some materials used in 3D printing dissolve in contact with water or alcohol. Others have strongly hydrophobic surfaces, which severely limit their application as potential materials.
- Durability during the measurement period.

In addition, the print itself had to reproduce the given object with the required accuracy, which is already dependent on the quality of the printer’s work.

Clear FLGPCL04 resin (light-curing resin) from Formlabs was selected for testing. The used printer was Form3, which is associated with low-force stereolithography (LFS) technology. The printing process itself, depending on the parameters, gives different results. The used resolution was 25 µm, i.e., the highest possible. The producer claims that the printer goes through rigorous factory calibration and that no on-site calibration is required.

**Figure 2:**
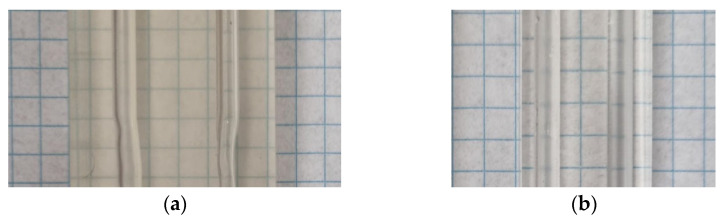
(**a**) Yellowing effect and (**b**) clear block.

UV radiation curing increases the hardness of the sample but also causes yellowing (Figure 2a). Shortening this time allows more transparent objects to be obtained but at the expense of hardness, thus affecting the possibility of polishing the outer layer (Figure 2b). In our tests, this time was in the range of 5–10 min. After printing, the model was cleaned of residual resin using isopropanol and distilled water, dried, and polished on the outer walls. Hand-sanding was performing using wet sandpaper with decreasing grit from P1000 to P12000. Inner walls made contact with the fluid with a similar refractive index so that imperfect surface structures could be omitted. In our print results, we did not observe any issues with the inner walls. The next step involved conversion, namely with a coating that protects against natural UV radiation, potentially causing slower degradation in the test object. The resin that was used has good parameters in terms of chemical resistance and physical strength. Unfortunately, the manufacturer does not provide a refractive index, which has to be measured and may also depend on the parameters of the received print. Experiments have also shown that during measurements, the inner walls become contaminated with a small deposit of seed particles, but it is possible to wash them out with an ethanol solution.

### 2.2. Liquid Solution (Blood-like Fluid)

The project assumed that the measurement conditions closely resemble the conditions that naturally occur in the human body. It is impossible to work with blood at this stage, but the most common substitute is a mixture of water and glycerin. Thus, a 45 wt% glycerin solution was used during the measurements. The viscosity of the solution at 24 °C was tested on an Anton Paar rotational rheometer. The viscosity obtained at the measurement temperature was 4.07 mPas. The density was set to 1113.8 kg/m^3^ according to the literature data [21]. Rhodamine-B-labeled poly(methyl methacrylate) particles, with a mean diameter of 10 µm and a maximum emission wavelength of around 580 nm, were used as seedling particles.

## 3. Experimental Setup

### 3.1. PIV System

**Figure 3:**
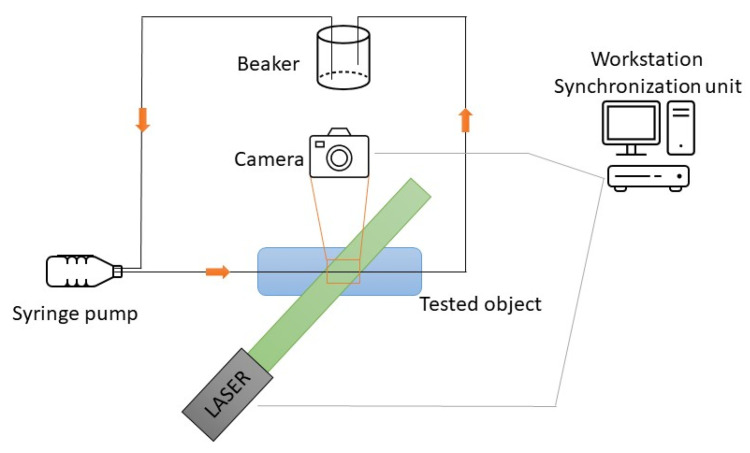
The main components of the research setup.

The experimental setup was designed specifically for the studied case. PIV experiments were conducted using a typical PIV system based on a double-pulsed 532 nm laser with a <10 ns pulse duration, where a laser beam was properly directed with mirrors to the light sheet optics. The time between pulses was selected according to the flow and the narrowing of the tested geometries. A double-frame camera was used with a resolution of 2048 × 2048 pixels, a 7.4 µm pixel size, and a 12-bit pixel depth. The configuration of the system was rather similar to macro-scale measurements with light sheet optics, unlike confocal illumination, which uses a microscope. For camera optics, a microscope with a long-pass filter that blocks <550 nm light (including laser light) was used. The 3D-printed object was placed horizontally on an XYZ translation stage with a micrometer screw that allowed for the object to be accurately positioned in relation to a field-of-view (FOV) camera. This also ensured stability between the microscope and the test object. Even small shocks, e.g., using an accidental table hit, generated small vibrations that were recorded with the camera and affected the measurement run. For flow visualization inside the geometry, polyamide particles, with a size distribution of 1–20 µm and with dissolved rhodamine type B, were used. Everything was managed using DynamicStudio v7 fluid measurement software and the TimerBox TTL synchronization unit. The main components of the research setup are shown in Figure 3.

**Figure 4:**
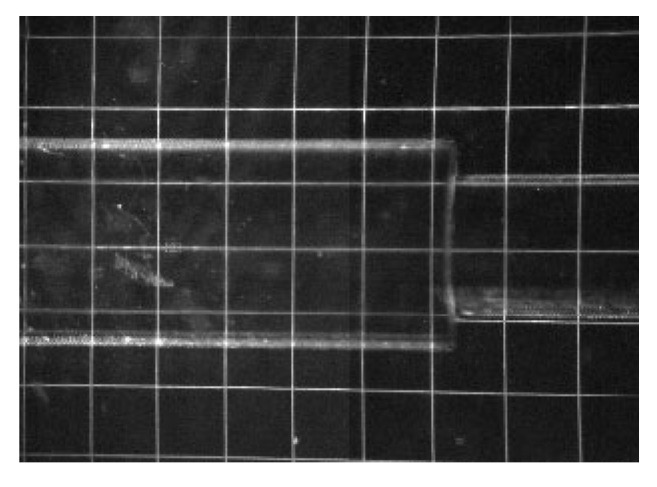
Calibration tool.

**Figure 5:**
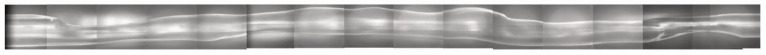
PIV field-of-view image without seeds.

The calibration tool was developed specifically for this experiment (Figure 4). As can be seen, the refractive index was not ideally matched, but it was still acceptable for this case since the first step involved finding a proper material. To improve the PIV accuracy in the area of the boundary layer, interrogation window masking was applied. The measurement started in the axis of the inlet pipe and continued along the vessel without changing the height perpendicular to the model axis. The base images from 16 consecutive exposures were stitched to visualize the entire vessel (Figure 5).

Our model had a 4.2 mm inlet diameter and a system resolution of about 3.3 × 3.3 µm per camera pixel. In our case, the only tool used to measure the actual size of the printed artery was the ruler from PIV software DynamicStudio v7. With that, we achieved an exact result of 4.2 mm ± 1% (indicating uncertainty from PIV calibration).

### 3.2. Flow Loop

Measurements with the water–glycerin particle solution were performed as a closed loop. First, the solution was aspirated into two 100 mL syringes using a syringe pump. The measured flow rate was constant, where the maximum chosen flow rate was the maximum capacity of the syringe pump at 300 mL/min. The two other tested flow rates were proportionally half (150 mL/min) and one-sixth (50 mL/min) of the maximum flow rate. With the maximum flow rate, the maximum pump operation time until the syringes were emptied was below 40 s. After considering the need for the buffer to obtain a constant, by developing a liquid velocity profile at the test side, this period was expected to reduce to less than 30 s. As a result, a 20 s measurement time was set for all flows with a measuring frequency of 7 Hz, and the results are satisfactory for this geometry (140 paired images in total). A summary of the flow conditions is shown in Table 1.

To stabilize the inlet stream, the fluid was delivered to the inlet of the pipe by a 30-cm-long straight tube with a diameter of 4 mm. From our calculations, the length with a maximum flow rate gives a fully developed profile.

## 4. Results

**Figure 6:**
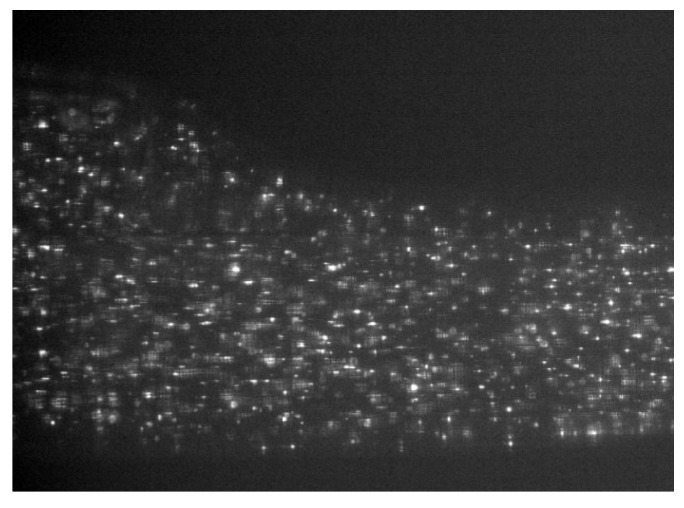
Raw PIV image from the measured object.

The photographs obtained using the camera during the measurement process are presented in Figure 6. The laser illumination appears to be homogeneous. It is easy to observe single-focused seed particles, but some that are blurred and unfocused are also visible. The image also shows a reduction in quality near the edges of the model.

For the PIV analysis, the Adaptive PIV method was used with the adaptive interrogation area (IA; ranging from 32 × 32 to 64 × 64 pixels) and a 50% overlap. After analysis, it turned out that the dominant IA had a size of 32 × 32 pixels with an average 0.005 particle per pixel and 2–3 particles per IA. No image corrections were necessary.

**Figure 7:**
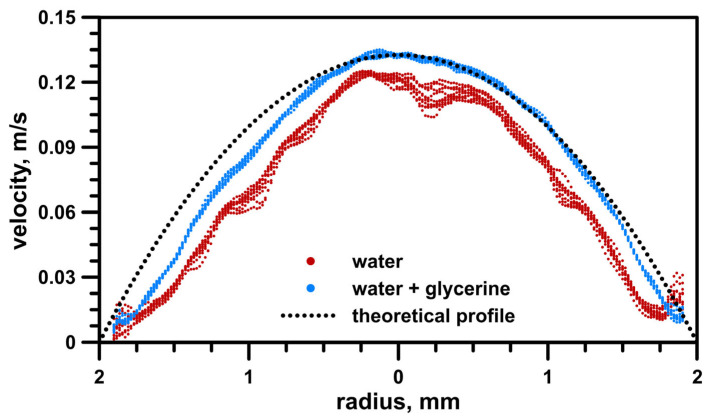
Axial velocity profile comparison of water flows, water + glycerin flows, and mathematical profile.

**Figure 8:**
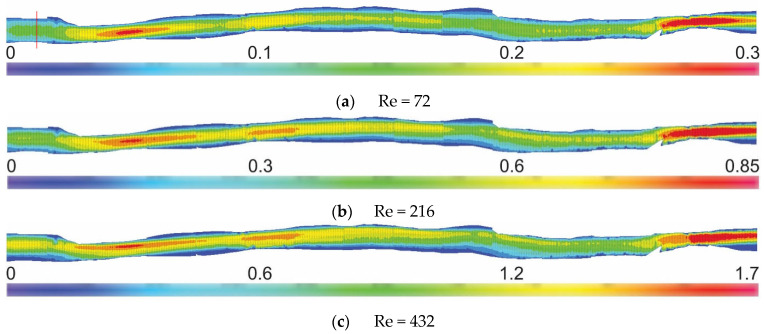
Vector statistics and velocity magnitudes (m/s) along the whole blood vessel for different Re numbers. The red line in (**a**) shows the place where the Reynolds numbers were calculated.

The distortion influence (due to different refractive indices) was tested, and the results are shown in Figure 7. Measurements were taken at the inlet just before the start of the geometry (the exact place is shown in Figure 8a). To compare the difference, clear water measurements were undertaken using the same settings. On the graph, we can see:

- Red dots—10 water profile plots of the vector statistic from 100 instantaneous maps acquired in the axis of the pipe;
- Blue dots—10 water + glycerin solution profile plots of the vector statistic from 100 instantaneous maps acquired in the axis of the pipe;
- Black line—theoretical parabolic laminar profile from the Navier–Stokes equation.

The results showed that the water–glycerin solution dramatically changed the values obtained compared to the flows of water alone. The deformation effect was greater when closer to the pipe walls. For both boundary layers, there were some velocity increases at the edges, which were much smaller for the water–glycerin solution than for water.

In Figure 8, the results of the stitched 2D PIV are presented. The real values of the vectors statistics along the whole geometry of arteries are shown with a Reynolds (Re) number of 3 at the inlet. The exact place where the Re number was calculated is marked in Figure 8a (red line). This is the same place where markings in Figure 6 were measured. The results show color-coded velocity vector maps of the entire 3D-printed vessel. The connections between consecutive images were seamless and consistent. We observed that the velocity values increased proportionally, while the structure changed typically and as expected. No boundary defects were noticed. Everything can be compared with CFD results due to the geometry mapping using a 3D printer.

## 5. Conclusions

This study aimed to find the best solution when using 3D printing technology to measure the geometry data of anatomical vascular models. The described methodology indicates the great potential of the selected 3D printing technology and material for printing difficult geometries. Though a previous study [19] reported the influence of print orientation on blurring reduction, we did not observe any correlations using an LFS 3D printer. The same conclusion was made in [20], where the same resin and 3D printer were used. However, we could easily observe the dependence of the quality of the outer surface and obtained images. Manual polishing is not practical for a higher number of models, as its accuracy and repeatability can be unrealizable. This part of the preparation of 3D-printed objects must be developed as a standard machine procedure. However, applying an additional layer that protects against natural UV radiation seems to be redundant with proper storage. There are other elements that can be improved to make the measurements more accurate and to more effectively reproduce natural conditions. Obtaining comparable refractive indices of the material and fluid is extremely important. One of the disadvantages of using a resin material for 3D printing is the relatively high refractive index (which is approximately 1.5 and is impossible to obtain with only a glycerin and water solution). Sodium iodide or other similar additions are required but with maintaining the condition of a liquid being blood-like fluid. In addition, the calibration tool has to be custom-designed and, even better, certified.

Expanding the research area to study paravalvular leaks via a 3D print has the major advantage of gel- or silicone-based phantoms. This paper confirms that cheap 3D printers can be used to obtain suitable models with satisfactory parameters. In addition, LFS seems to be a good choice.

Further work should aim to measure longer flow periods and flows for larger Re numbers for some geometries. We plan to apply our findings in future studies that will include measurements of paravalvular leaks in the human heart. Hence, future work with time-resolved stereo-PIV is planned to resolve the 3D nature of the flow in natural heart pulse conditions.

## Figures and Tables

**Table 1:** Table of flow conditions.

Inlet Diameter (mm)	Flow Rate (mL/s)	Reynolds Number	Inlet Velocity Amplitude (m/s)
4.2 mm ± 1%	0.83	72	0.14
4.2 mm ± 1%	2.5	216	0.51
4.2 mm ± 1%	5	432	1.25

## Data Availability

The data presented in this study are contained within the article.
